# Statistical data transformation in agrarian sciences for variance analysis: a systematic review

**DOI:** 10.12688/f1000research.144805.1

**Published:** 2024-05-08

**Authors:** Jhennifer Nascimento, Jonas Silva, Rodrigo Cupertino Bernardes, Guilherme Costa, Paulo Emiliano

**Affiliations:** 1 Department of Statistics, Federal University of Vicosa, Viçosa, State of Minas Gerais, Brazil; 2 Department of General Biology, Universidade Federal de Vicosa, Viçosa, State of Minas Gerais, Brazil; 3 ICTP South American Institute for Fundamental Research and Instituto de Fíica Teórica, São Paulo State University, São Paulo, State of São Paulo, Brazil

**Keywords:** Data manipulation, Systematic literature review, Agricultural sciences, ANOVA, Descriptive analysis

## Abstract

In statistical analyses, a common practice for enhancing the validity of variance analysis is the application of data transformation to convert measurements into a different mathematical scale. This technique was first employed in 1898 by Edgeworth and remains relevant in current scientific publications despite the proliferation of more modern and advanced techniques that obviate the need for certain assumptions. Data transformations, when appropriately used, can make the model error terms approximate a normal distribution. It is also possible to use the technique to correct the heterogeneity of variances or to render an additive model, ensuring the validity of the analysis of variances. Given that this technique can be hastily applied, potentially leading to erroneous or invalid results, we conducted a systematic literature review of studies in the field of agrarian sciences that utilized data transformations for the validation of analysis of variances. The aim was to check the transformations employed by the scientific community, the motivation behind their use, and to identify possible errors and inconsistencies in applying the technique in publications. In this study, we identified shortcomings and misconceptions associated with using this method, and we observed incomplete and inadequate utilization of the technique in 94.28% of the analysed sample, resulting in misguided and erroneous conclusions in scientific research outcomes.

## 1. Introduction

The analysis of variance (ANOVA) is a method used to test the hypothesis of statistical equality among a set of population means, which is a superior option to the t-test due to the increased probability of committing a type I error when using it for comparing multiple means, as stated by Mohr. ^1^ To validate the ANOVA analysis, assumptions such as additivity of treatment and environmental effects, independence, homoscedasticity of variances, and normality of experimental errors need to be satisfied, as pointed out by Cochran. ^2^ Data transformations can be applied to validate the analysis when these assumptions are unmet.

Initially proposed by Edgeworth ^3^ and referred to as the Translation Method, data transformations emerged from the need to relate observed distributions to the normal distribution. According to Bartlett, ^4^ the usual purpose of transformation is to change the scale of measurements to make the ANOVA analysis valid. However, a transformation is successful only if it satisfies the requirements of variance homogeneity, additivity structure, and error normality through the same transformation, as stated by Atikinson. ^5^

After any transformation, verifying whether it has improved the data distribution is essential, as highlighted by Quinn and Keough. ^6^ Besides that, data transformation provides an accessible solution to avoid non-normal error distributions, as stated by Pierre et al., ^7^ allowing for easy analyses by applying linear models. However, researchers must exercise caution when using transformations, as they can have contrary effects, Rupert. ^8^

Despite frequently using data transformations in scientific literature, they receive criticism from theoretical and practical perspectives, Oliveira et al. ^9^ One of the main criticisms of data transformations is the potential to alter the interpretation of results, Box and Cox. ^10^ According to these authors, data transformation can change the focus of the study, leading to different conclusions than those obtained from the original data. Additionally, some transformations may not be readily interpretable, hindering the communication of results due to the transformed scale. Another criticism is that transformations can affect the robustness of ANOVA, resulting in false positives or negatives, Hocking. ^11^ Therefore, carefully considering the implications of any data transformation before applying it in a variance analysis is crucial.

Due to the large volume of studies in agrarian sciences that conduct variance analyses and can make use of various data transformations to validate them, a systematic literature review is a valuable approach to identifying subject trends and determining whether there is proper utilization of transformation techniques to ensure reliable and valid results of variance analysis. According to Khan, ^12^ a systematic review involves systematically collecting and examining published works to extract relevant statistics and information from the selected studies. On the other hand, meta-analysis is the quantitative aspect of synthesizing the analysed works, allowing for a numerical summary of the observed results.

In this context, we conducted a systematic literature review of published works in agrarian sciences that employ data transformations to validate variance analyses. We performed a detailed analysis of various publications that utilized this technique to identify the most common transformations adopted by the scientific community and the motivations behind their use. This study also identified shortcomings and misconceptions associated with applying this method. The results of the analysed sample revealed an inadequate application of the technique by the scientific community, highlighting the need for enhanced methodological rigor in such analyses by researchers.

## 2. Methods

### 2.1 Data collection

The systematic literature review process adopted in this study followed the guidelines of PRISMA (Preferred Reporting Items for Systematic Reviews and Meta-Analyses), developed by Moher et al. ^13^ These guidelines describe the stages of identification, screening, eligibility, and inclusion, ensuring the results’ validity and reliability.

In the identification stage, studies of interest are located in the databases, followed by selecting and classifying works based on titles and abstracts in the screening stage. In the eligibility stage, we evaluate the full text of the selected articles from the previous stage. In the inclusion stage, relevant information is systematically extracted based on predefined topics.

However, our methodology adapted to these guidelines in the screening phase. Instead of analysing only the articles’ abstracts, we downloaded the selected articles in the identification stage. We searched for specific terms in the full text of these works to select the articles that would proceed to the eligibility and inclusion stages. This strategy was employed because crucial information about the transformations is sometimes present in the abstracts, title, and methodology. This way, the information of interest would be noticed.

*2.1.1 Identification stage*

To identify articles in the field of agricultural sciences that have conducted variance analyses, we conducted comprehensive searches on academic platforms such as Scopus and Web of Science, using keywords such as “variance analysis,” “analysis of variance,” and “ANOVA.” We narrowed our search to articles published exclusively in English, the global language of academia and research. This approach ensures a uniform and accessible foundation for the chosen studies.

Due to the absence of specific filters for agricultural sciences in the platforms used, we employed different refinement approaches for each platform. In Scopus, under the “subject area” tab, we filtered by “Agricultural and Biological Sciences,” excluding journals unrelated to agricultural sciences. In Web of Science, under the “Web of Science Categories” tab, we filtered by “Agronomy,” “Agriculture Dairy Animal Science,” “Agriculture Multidisciplinary,” “Agricultural Engineering,” and “Agricultural Economics Policy.” The last search for each source was conducted on May 14 ^th^.

*2.1.2 Screening stage*

Due to the high quantity of files to be downloaded and analysed, we only worked with open-access files. We exported these files in the RIS format to Rayyan, a web application developed by the Qatar Computing Research Institute for systematic reviews. We organized the files according to the publication year and manually excluded duplicates to prevent unnecessary file downloads.

We utilized the “pyautogui,” “openly,” and “pandas” libraries to develop a Python automation that downloads the files in pdf format directly from Scopus and Web of Science platforms. The ones not downloaded through automation were obtained manually.

Subsequently, we employed the “pypdf4” library to develop a Python search code to search for the terms “data were transformed,” “transformation of the data,” and “data transformation” within the full texts of the works, aiming to reduce the number of articles required for reading and analysis.

Python is freely available software accessible at https://www.python.org/, and the Python code can be found at https://github.com/ghscosta/data-transform for anyone interested in replicating the procedures outlined in this article.

*2.1.3 Eligibility and inclusion stages*

During the eligibility stage, two reviewers examined the complete texts of the articles resulting from the screening process and extracted relevant information in the inclusion phase. We carried out these two stages simultaneously. From the articles that underwent the initial filtering process, we collected the following information: reference number for article identification, title, journal, year of publication, authors, utilization of analysis of variance in the methodology, employed design, verification of ANOVA assumptions, type of applied transformation, rationale for the adoption of the transformation, re-evaluation of assumptions after data transformation, scale of interpretation for results, study element in the experiment, and impact factor of each journal. The description of each variable can be accessed in Table 1, available at https://rpubs.com/JhenniferNascimento/table and https://zenodo.org/records/10757759. We discarded articles that did not provide this information and considered only those containing the pertinent details for the descriptive analysis.

### 2.2 Descriptive analysis

To present the collected data and potential relationships among variables, we employed bar graphs, pie charts, and Sankey diagrams for representing and analysing the information obtained from the sampled articles. We manually tabulated the data, and a concise summary is provided in our extended data, accessible in Table 2 at https://rpubs.com/JhenniferNascimento/table and https://zenodo.org/records/10757759.

Pie and bar charts were used to illustrate the eligibility criteria for determining the inclusion of studies in each synthesis, along with the percentages of discarded and included studies. Sankey diagrams were employed to depict key variables and elucidate potential relationships among them.

## 3. Results

### 3.1 Data Collection

*3.1.1 Identification stage*

The search for the terms “variance analysis,” “analysis of variance,” and “ANOVA,” which we conducted on the Web of Science and Scopus platforms, resulted in retrieving $8,460$ files on the former and 8,695 on the latter. Out of this total, 3,615 files from Web of Science and 3,529 from Scopus were available in open access. Consequently, during the identification stage, we obtained a combined set of 7144 articles published between 1961 and 2023 from both platforms.

*3.1.2 Screening stage*

After the removal of duplicates, 6,077 articles remained for downloading. We downloaded 5,032 articles through automation, leaving 1,045 for manual download. Despite applying the open access filter on both platforms, we could not access 52 of the articles earmarked for manual downloading, resulting in a total of 6,025 articles successfully downloaded.

By searching for the terms “data were transformed,” “transformation of the data” and “data transformation” within the full text of the 6,025 articles, we narrowed down the number of articles requiring scrutiny from 6,025 to 565 for analysis in the upcoming stages.

*3.1.3 Eligibility and inclusion stages*

We read and collected relevant information from 565 articles, resulting from the previous stages. Among these, 506 articles were discarded, with details about the discards presented in the descriptive analysis. We effectively collected information from only 59 articles relevant to our search.

Some of the tabulated articles conducted more than one experiment in their work or used more than one transformation, thus leading to more than one variance analysis. Each distinct experiment and transformation were treated as separate observations to ensure correct counting. As a result, the sample of 59 articles generated 70 tabulated observations. The study characteristics are presented in Table 2, available at https://rpubs.com/JhenniferNascimento/table.

### 3.2 Descriptive analysis

**Figure 1.**
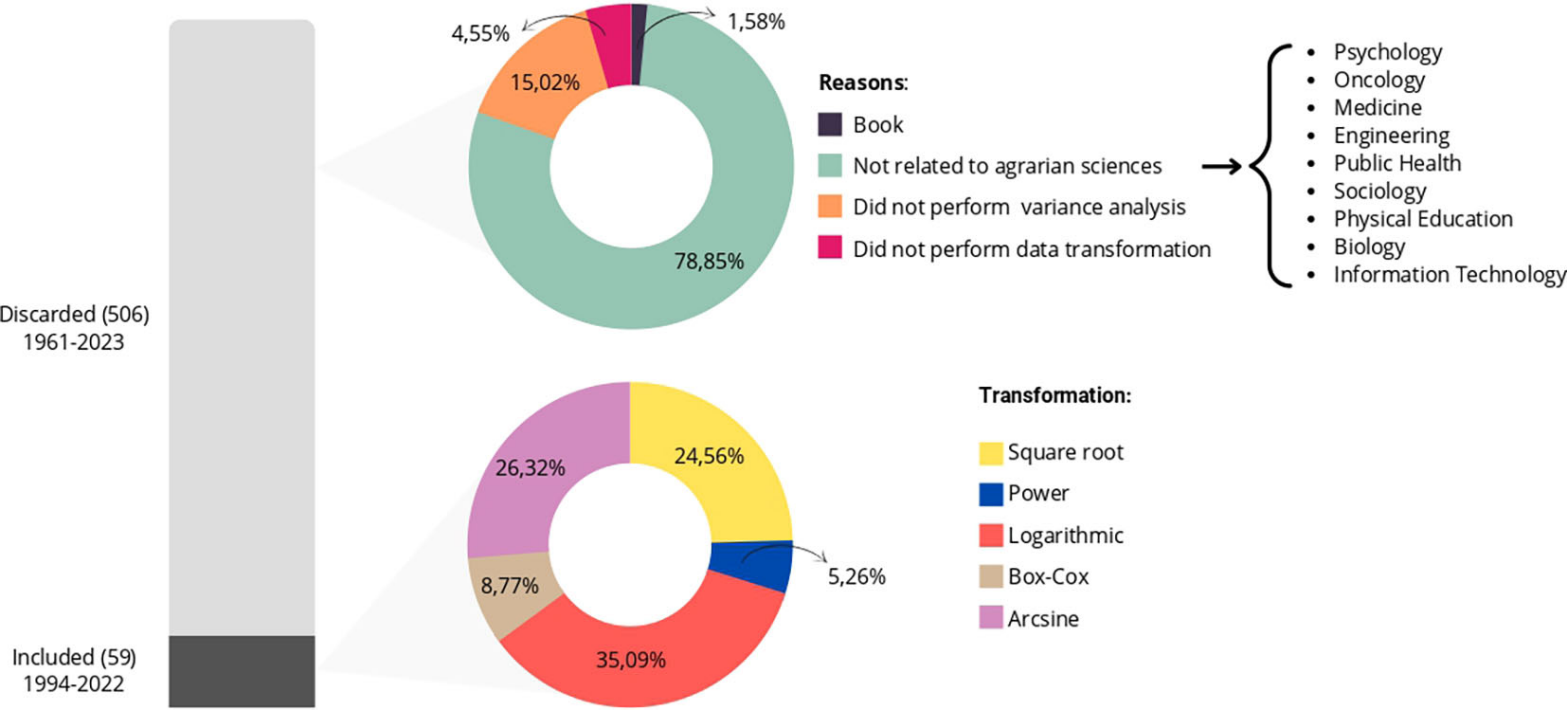
A descriptive analysis of the included and discarded articles, considering the field of study of the works and the types of transformations found in them.

Figure 1 presents the quantities of articles discarded and included for tabulation, where the stacked bars visually show the total of 506 and 59 articles discarded and included in the final sample, respectively. On the other hand, the first pie chart presents the reasons used for the discard, and the second one shows the transformations used for the validation of the analysis of variances.

In the first circular sector, it is evident that we discarded approximately 78% of the articles from fields like medicine, engineering, psychology, and physical education because they were outside the field of agrarian sciences. We discarded approximately 15% of the articles for not containing variance analysis, 4.55% for not using data transformation, and just over 1% were books and, therefore, incompatible with the inclusion criteria.

**Figure 2.**
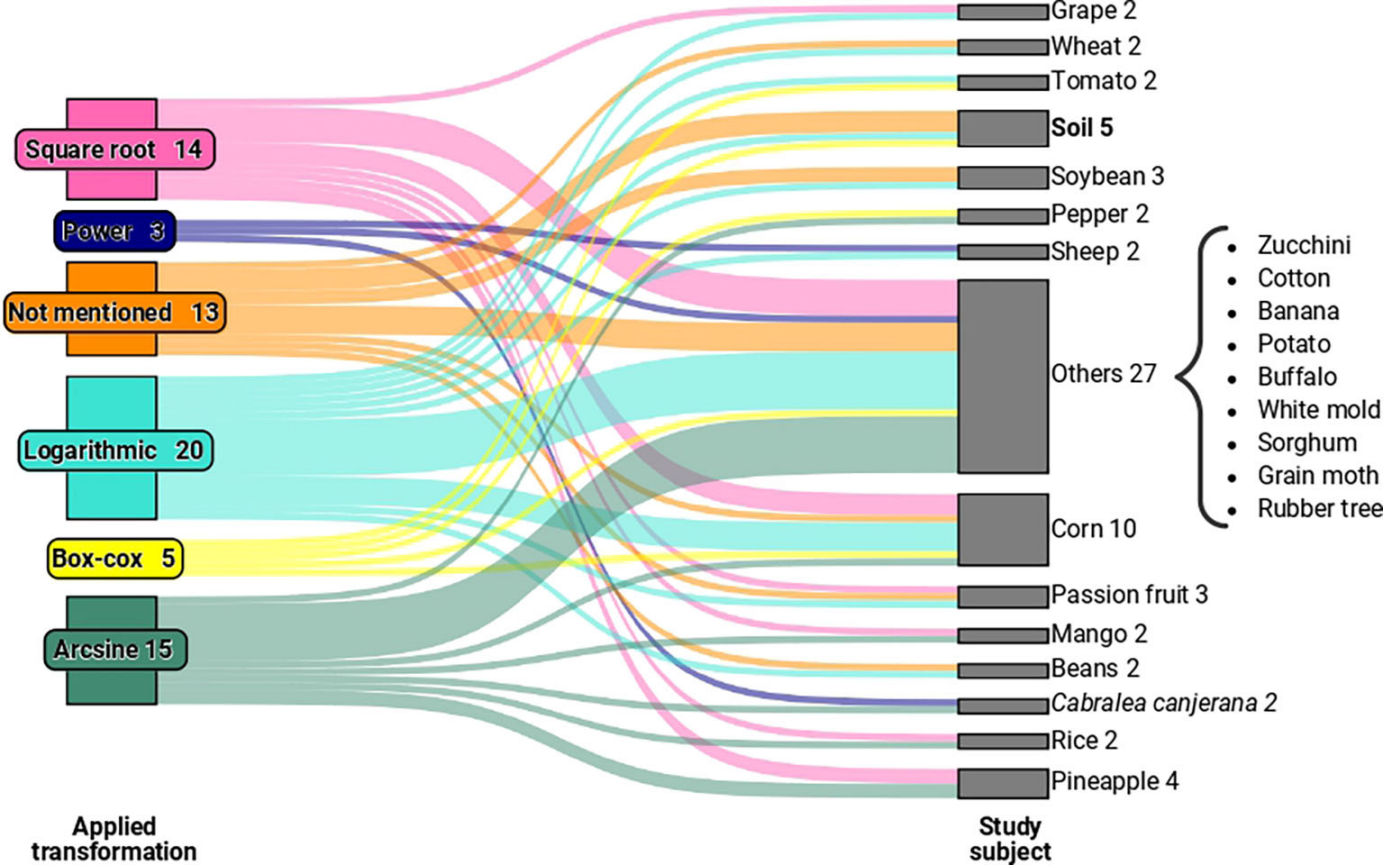
A Sankey diagram representing the relationship between the transformations used in the evaluated studies and the study elements of the experiments. In the “Other” category, we have study elements that had only one occurrence in the sample.

In the second circular sector of Figure 1, which relates to articles with relevant information for our study published between 1994 and 2022, we observed five types of transformations. These include logarithmic, square root, Box-Cox, power, and arcsine transformations, used to validate variance analysis in experiments involving crops such as corn, rice, passion fruit, and others, as illustrated in Figure 2. We grouped categories with only one occurrence as “other” for the “study element” variable.

**Figure 3.**
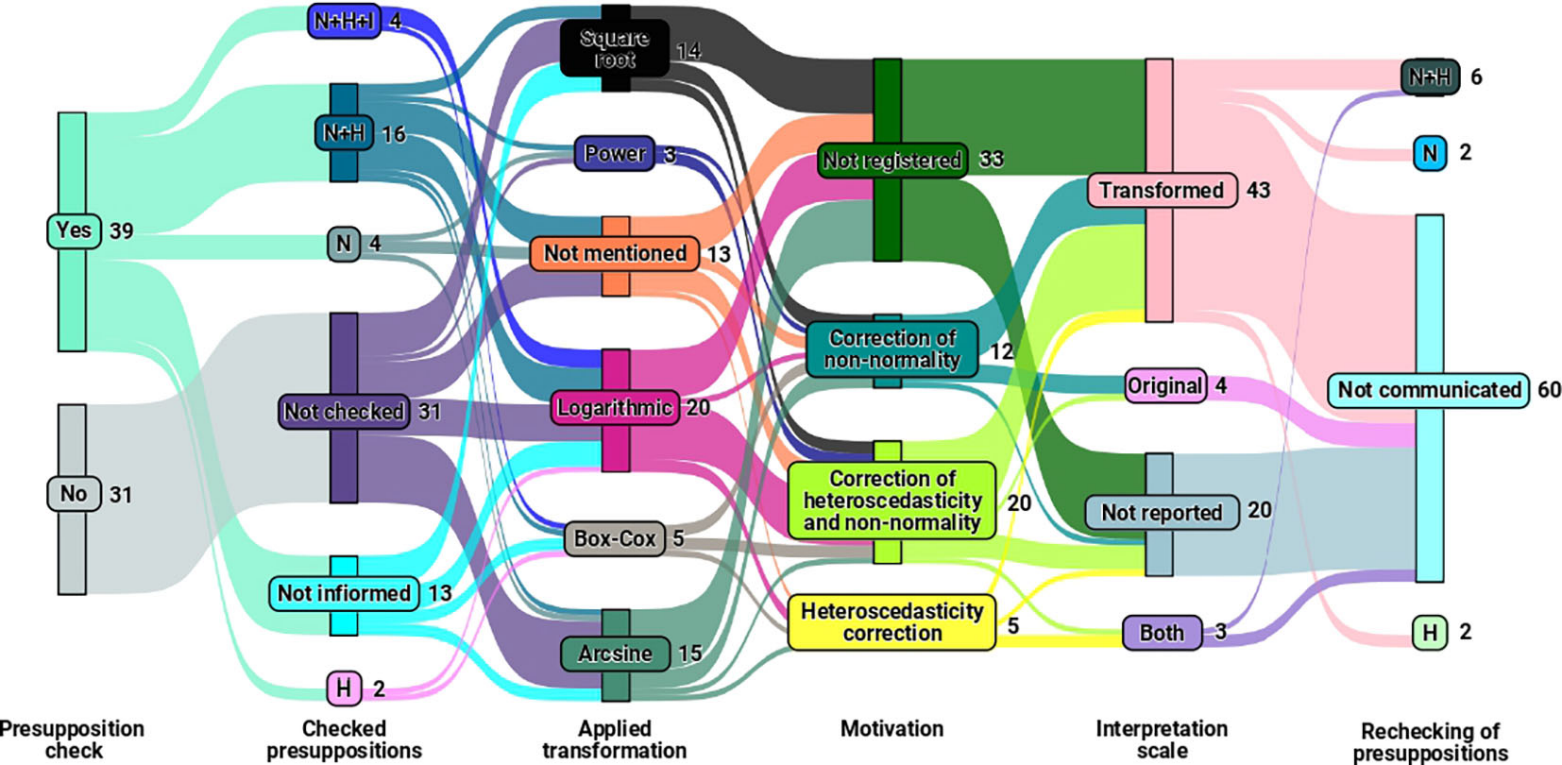
Sankey diagram illustrating the relationships between the transformations used, the verification of assumptions (N for normality, H for homogeneity of variances, N+H for normality and homogeneity of variances, and N+H+I for normality, homogeneity of variances, and independence), the motivation for using these transformations, the scale of interpretation, and the re-verification of assumptions.

In addition to examining the relationship between the study elements of the experiment and the transformations used, we investigated using Figure 3 to explore the interactions and connections among the transformations. We also verified assumptions, assessed the motivation for employing these transformations, considered the interpretation scale, and reevaluated the assumptions of analysis of variance after applying the transformation.

As a result, Figure 3 highlights that transformations were applied within the final sample with ( 55.71% ) and without ( 44.28% ) the verification of analysis of variance assumptions. Among the sampled studies that checked the assumptions, 66.66% verified at least one of the three assumptions (normality, homogeneity, and independence of residuals) before applying the transformation, while 33.33% mentioned the verification of assumptions but did not specify which the checked assumption.

Among the observed transformations in the sample (as illustrated in Figure 3), logarithmic ( 28.57% ), arcsine ( 21.42% ), and square root ( 20% ) transformations were the most used, regardless of whether checked assumptions. At the same time, 18.57% did not specify the transformation used.

Figure 3 also reveals the presence of articles that did not verify assumptions, did not specify the transformation used, did not record the rationale behind the choice of transformation, and did not report whether the meted assumptions of analysis of variance after the application of the transformation. Only 5.71% of the studies conducted verification of normality, homogeneity, and independence of residuals assumptions and adopted transformations with plausible justifications and motivations, illustrating the technique’s incomplete and potentially incorrect application in 94.28% of the considered studies.

Finally, Figure 3 also provides relevant information regarding the interpretation approach adopted in the studies. Most articles interpreted their results on the transformed scale, considering the data after applying transformations. However, some studies mentioned inverse transformation to revert the data to the original scale before interpretation, which is an incorrect practice. As for the reevaluation of assumptions after the implementation of transformations, most studies did not mention this step.

## 4. Discussion

### 4.1 Data collection

Executing the steps proposed by PRISMA (identification, screening, eligibility, and inclusion), with the implemented adaptations, presents an innovative approach to searching and selecting articles for this study. The direct search for the terms of interest in the full text represented a bold strategy, one that could potentially serve as a valuable technique for future systematic reviews, particularly when researchers seek information that may not be explicitly stated in the title or abstract, as was the case in our study.

The screening phase, implemented with automated article downloading and the search for terms in the complete text, programmed in the Python language, efficiently directed us to the most relevant articles with minimal manual effort required. Although some articles required manual downloading, we significantly reduced the overall effort.

During the eligibility and inclusion phases, we identified and selected articles that reported essential information about the implementation of data transformations to validate the analysis of variance. We notice that many of these studies did not unsurprisingly document several relevant pieces of information.

### 4.2 Descriptive analysis

As depicted in Figure 1, a large portion of the articles was discarded, which is common in systematic literature reviews, as pointed out by Gerstner. ^14^ According to the authors, essential information is often omitted in scientific papers, leading to the exclusion of these studies during the systematic review or meta-analysis stage. In our study, only 10% of the sample provided the information of interest, with the majority of exclusions occurring because they were unrelated to the agrarian sciences field. This result was surprising since we expected to obtain a more representative sample of the field due to the filtering criteria employed on the platforms.

When examining the transformations employed in the studies concerning the analysed elements in the experiments (as exemplified in Figure 2), no specific pattern emerged that linked a particular type of element to a specific transformation. By expanding the sample, it is conceivable that we may eventually discern a relevant pattern between the types of elements used in experiments and their interaction with specific transformations.

Regarding the interactions and connections of the variables presented in Figure 3, concerns arise about the validity of the analyses conducted in studies where 55% of the sample did not verify essential assumptions (normality, homogeneity, and independence of residuals) and opted for data transformations. The practice of transforming data without first determining whether it is necessary for the analysis compromises the interpretation and reliability of the results obtained.

On the other hand, the finding that 26 out of 39 articles that effectively assessed at least one of the three assumptions before applying transformations (equivalent to 66.66% ), representing only 37.14% of the total sample (of 71 articles), raises questions about the awareness and statistical rigor of authors in handling such data analysis. In contrast, the 33.33% of articles mentioning assumption verification without specifying which ones indicate a need for more transparency in statistical analysis practices.

The distribution of data transformations in the sample draws attention to the prevalence of logarithmic, arcsine, and square root transformations, regardless of assumption verification. However, the omission of transformation cases raises concerns about research transparency and reproducibility, underscoring the need for comprehensive documentation of methods to ensure accurate evaluation and replicability.

The finding that only a tiny portion of the studies adopted transformations with plausible justifications after verifying the assumptions of normality, homogeneity, and independence of residuals, coupled with the presence of studies that did not verify assumptions, did not mention transformations used and did not document justifications for their choices, raises questions about the statistical rigor of authors in conducting such analyses. It highlights the need for greater statistical rigor within the academic community and more explicit guidelines for conducting robust and transparent statistical analyses, which ensure reliable and meaningful results for advancing research.

Furthermore, the highlighted findings also raise essential considerations about academic journals’ peer review and publication process. The journal with the highest impact factor in the sample includes one of the articles that did not verify statistical assumptions, omitted details about applied transformations, and failed to document the rationale behind their choices. Accepting works with such characteristics impacts the quality of scientific literature and compromises the integrity of presented results and the correct interpretation of conclusions.

Therefore, readers of these works often need more evidence to determine the reliability of the executed analysis and the result’s veracity, having to rely on the content presented without the opportunity to develop their conclusions based on the analyses. It shows the importance of a complete approach in describing the procedures used in scientific works that are published and that journals need to be more careful, especially when it comes to implementing this methodology that is so old and used.

Regarding aspects related to result interpretation and the methodological approach adopted in the studies in our sample, the observation that most articles chose to interpret their results on the transformed scale points to an ordinary and correct practice in the literature. However, returning the data to its original scale before interpretation raises a discussion about the validity of this approach, as it can distort the correct interpretation of results and potentially lead to erroneous conclusions. For the articles interpreted in both scales, those made an unnecessary effort. Interpreting must always occur on the transformed scale.

Finally, another relevant point is the lack of mention of reevaluating assumptions after applying transformations. It raises questions about researchers’ awareness of the importance of verifying whether the assumptions of analysis of variance are met even after data transformation. Omitting this step can compromise the reliability and validity of conclusions. The documentation and detailed description of methods used in all scientific research is crucial, especially in the field of agricultural sciences, which was the focus of our study.

## 5. Conclusions

We introduced a novel approach to screening systematic literature reviews, which combines downloading and searching for the terms of interest directly within the full-text articles. This technique proves valuable, mainly when research of interest is not readily available in titles or abstracts.

The descriptive analysis of transformations used in the articles revealed the prevalence of logarithmic, arcsine, and square root transformations. It became evident that verifying assumptions before applying transformations was only consistently conducted in some cases, emphasizing the need for a more detailed and consistent approach in research planning and reporting procedures.

Interpreting results on the transformed scale was the most common approach, although some studies adopted reverse transformations to return data to the original scale. However, the omission of analysis details underscores the need for greater clarity and consistency in documenting the procedures employed.

Given our focus on the field of agricultural sciences and the identified inconsistencies in the technique’s application, questions arise regarding the appropriate use of this method in other areas of knowledge.

## Data Availability

All underlying data are available as part of the article and no additional source data are required. Zenodo: Extended data for ‘Statistical data transformation in agrarian sciences for variance analysis: a systematic review’, https://doi.org/10.5281/zenodo.10519177. ^15^ Zenodo: PRISMA checklist and flow diagram for ‘Statistical data transformation in agrarian sciences for variance analysis: a systematic review’, https://doi.org/10.5281/zenodo.10758186. ^16^ Data are available under the terms of the Creative Commons Attribution 4.0 International Public License (CC-BY 4.0 International).

## Software availability

Source code available from: https://github.com/ghscosta/data-transform/tree/v0.2.

Archived software available from: https://doi.org/10.5281/zenodo.10519177. ^15^

License: GNU General Public License v3.0.
