# Supplementary material for: Statistical data transformation in agrarian sciences for variance analysis: a systematic review
Source: F1000Res. 2024 Jul 12;13:459. Originally published 2024 May 8. [Version 2] doi: 10.12688/f1000research.144805.2 (PMC11564925; doi:10.12688/f1000research.144805.2)
Supplement: Peer review history

# Reviewer report: Reviewer response for version 2

**DOI:** 10.5256/f1000research.168986.r335944

**Published:** 2024-11-14

**Authors:** Juan Burgueno

**Affiliations:** 1 Biometrics and Statistics Unit, CIMMYT, El Batan, Estado de México, Mexico

The topic of the article is of interest to a broad audience and the main conclusions could be considered by journal editors to improve scientific communication. The review was done accordingly with the best practices.

 There are some elements in the article which the authors omitted, and it would be good to include. For a better comprehension of the problem and understanding of the results.

 A more detail of the ANOVA assumptions must be incorporated. Nothing is said about the robustness of ANOVA against non-normality. ANOVA works well if experimental error distribution is more less symmetric. In my understanding, independence of errors is reached by randomization and there is no way to assess it using residuals since they are not independent because they are calculated from the same data using estimated effects. I think. that the authors are talking about independence against estimated values or level of response which is more associated with lack of homoscedasticity of variances.

 A second point is about two sources of bias in the results and conclusions that must be clearly stated. The authors use articles in which transformation was used but they do not analyze the 506 articles in which it was not used. Did transformation not use because it was not necessary or because the authors omitted it? It is remarkably interesting to know how many times transformation was ignored. A second source of bias is the alternative use of articles and “experiments,” some authors are considered more than once in the last case. An author who did use adequately transformation or not will do the same for other experiments biasing the result if it is considered twice. It is fine to include both analyses, but the text is not clear which one is interpreted in the conclusions.

 In the literature you can find recommendations of specific transformations for specific variables, however the authors do not mention in the paper which kind of variables were transformed or not. It is not the same to transform percentages which came from counting than percentages calculated from continuous variables. Knowing which variables and how they were measured would be a good improvement to the article.

 The Sankey diagrams are nice and full of colors, but difficult to read. It is difficult to identify numbers by the thickness of the “rivers” and some number cannot be read because the color. A table with the number could be a better option.

 In Figure 1, two articles are missing.

 In Figure 2, a new transformation group is included, "Not mentioned" with thirteen articles. Why is it not in Figure 1? Why should you include in the study a transformation that cannot be evaluated? It is not clear what the numbers are in the figure, they do not match with numbers in the text and figure 1. In the text the authors move from Figure 1 to Figure 2 which use different data, without explanation.

 An interesting result that the authors do not discuss is that no articles before 1994 were included in the analysis. Why? Is it just causality?

 The authors conclude: “When examining the transformations employed in the studies concerning the analyzed elements in the experiments (as exemplified in Figure 2), no specific pattern emerged that linked a particular type of element to a specific transformation. By expanding the sample, it is conceivable that we may eventually discern a relevant pattern between the types of elements used in experiments and their interaction with specific transformations” The number of articles and the considerable number of "elements" make so difficult to conclude about this in any way.

Are the rationale for, and objectives of, the Systematic Review clearly stated?

Yes

Is the statistical analysis and its interpretation appropriate?

Partly

If this is a Living Systematic Review, is the ‘living’ method appropriate and is the search schedule clearly defined and justified? (‘Living Systematic Review’ or a variation of this term should be included in the title.)

Yes

Are sufficient details of the methods and analysis provided to allow replication by others?

Partly

Are the conclusions drawn adequately supported by the results presented in the review?

Partly

Reviewer Expertise:

Statistics and Biometrics

I confirm that I have read this submission and believe that I have an appropriate level of expertise to confirm that it is of an acceptable scientific standard, however I have significant reservations, as outlined above.

# Reviewer report: Reviewer response for version 2

**DOI:** 10.5256/f1000research.168986.r302315

**Published:** 2024-07-23

**Authors:** Pratheesh P Gopinath

**Affiliations:** 1 Kerala Agricultural University, Kerela, India

The explanation given by the authors were acceptable and no further comments.

Are the rationale for, and objectives of, the Systematic Review clearly stated?

Partly

Is the statistical analysis and its interpretation appropriate?

Partly

If this is a Living Systematic Review, is the ‘living’ method appropriate and is the search schedule clearly defined and justified? (‘Living Systematic Review’ or a variation of this term should be included in the title.)

Not applicable

Are sufficient details of the methods and analysis provided to allow replication by others?

Yes

Are the conclusions drawn adequately supported by the results presented in the review?

No

Reviewer Expertise:

Design of Experiments, AI, R shiny, R, Agricultural Statistics, Social Science, Climate Statistics

I confirm that I have read this submission and believe that I have an appropriate level of expertise to confirm that it is of an acceptable scientific standard.

# Reviewer report: Reviewer response for version 1

**DOI:** 10.5256/f1000research.158652.r275867

**Published:** 2024-11-14

**Authors:** Manoj Kumar Sharma

**Affiliations:** 1 Sri Karan Narendra Agriculture University, Jobner, India

Comments on the paper “Statistical data transformation in agrarian sciences for variance analysis: a systematic review”.

 The author (s) have nicely written the proposed paper. It is a unique type of work. This should be widely popularized. The suggestions and comments from my side are given below impactful presentation. 

 1.The introduction and methods are nicely presented.

 2.In the results section the findings are presented only in the form of data-oriented figures. In my point of view, the finding data should also presented in tabular form.

 3.In the discussion part some of the results are also presented. So these results should also shifted to the results section.

 4.The Conclusion section is nicely presented.

 The author (s) should be asked to revise the paper as per my suggestions mentioned above. This paper may be accepted after minor revision.

Are the rationale for, and objectives of, the Systematic Review clearly stated?

Yes

Is the statistical analysis and its interpretation appropriate?

Partly

If this is a Living Systematic Review, is the ‘living’ method appropriate and is the search schedule clearly defined and justified? (‘Living Systematic Review’ or a variation of this term should be included in the title.)

Yes

Are sufficient details of the methods and analysis provided to allow replication by others?

Yes

Are the conclusions drawn adequately supported by the results presented in the review?

Yes

Reviewer Expertise:

Applied statistics

I confirm that I have read this submission and believe that I have an appropriate level of expertise to confirm that it is of an acceptable scientific standard.

# Reviewer report: Reviewer response for version 1

**DOI:** 10.5256/f1000research.158652.r287787

**Published:** 2024-06-25

**Authors:** Pratheesh P Gopinath

**Affiliations:** 1 Kerala Agricultural University, Kerela, India

The work is commendable for its initiative and effort to study data transformation practices in agrarian sciences. It sheds light on the significant issue of improper application of transformations, which can undermine the validity of statistical analyses.

 The importance of correctly applying transformations cannot be overstated, as it directly impacts the reliability of research findings. The study is particularly interesting as it addresses the critical aspect of when and how data transformations should be applied in statistical analyses. This is of paramount importance in agrarian sciences, where the choice of transformation can significantly influence the results. But the study lacks specificity.

 While the study does highlight the prevalence of improper transformation applications, it lacks a clear linkage to the motivations behind these practices. Researchers often follow established precedents without conducting normality and homogeneity tests, which is a critical gap that this review should address more explicitly, for example entomology studies mainly employs logarithmic and square root etc.

 A more detailed field-wise classification of the reviewed studies would enhance the usefulness of this study. Identifying which transformations are used in specific subfields, such as crop science, soil science, and entomology, would provide more actionable insights for researchers. A table showcasing the types of transformations used and their corresponding experimental situations would greatly enrich the paper. This classification would offer a practical guide for researchers on appropriate transformations for their specific research contexts.

 The presentation of results could be improved. For instance, in pie diagrams instead of decimals commas are used. Clarity of Sankey diagram in conceiving information is also questionable.

 While descriptive analysis is a useful starting point, it is not sufficient on its own to reveal underlying patterns and issues. The study would benefit from a more in-depth statistical analysis to identify trends and common pitfalls in the application of data transformations.

 The conclusion part needs to be more specific rather than stressing the novelty of data collection method the concentration can be on the analysis of trend of employing transformations and important conclusion based on that and suggestions.

Are the rationale for, and objectives of, the Systematic Review clearly stated?

Partly

Is the statistical analysis and its interpretation appropriate?

Partly

If this is a Living Systematic Review, is the ‘living’ method appropriate and is the search schedule clearly defined and justified? (‘Living Systematic Review’ or a variation of this term should be included in the title.)

Not applicable

Are sufficient details of the methods and analysis provided to allow replication by others?

Yes

Are the conclusions drawn adequately supported by the results presented in the review?

No

Reviewer Expertise:

Design of Experiments, AI, R shiny, R, Agricultural Statistics, Social Science, Climate Statistics

I confirm that I have read this submission and believe that I have an appropriate level of expertise to confirm that it is of an acceptable scientific standard, however I have significant reservations, as outlined above.

# Response

**Published:** 2024-07-10

**Authors:** Jhennifer dos Santos Nascimento

**Affiliations:** Statistics, Federal University of Vicosa, Viçosa, State of Minas Gerais, Brazil

Reviewer 2: Pratheesh P Gopinath, Kerala Agricultural University, Kerela, India.

 We appreciate the positive comments on our study. We agree that the correct application of data transformations is crucial for the reliability of research results in agricultural sciences. As discussed in paragraphs 6, 8, and 10 of section 4.2 of our article, we believe that the inadequate application of statistical techniques, including data transformations, often stems from a lack of statistical rigor in the academic community and the absence of explicit guidelines for conducting robust and transparent statistical analyses. Scientific journals should require authors to provide a complete and detailed description of the statistical procedures used in their work, as accurate documentation and description of methods are fundamental for the replicability and validity of research.

 Our study could have addressed the motivations behind inadequate data transformation practices more explicitly. As noted in our study, many researchers follow established precedents without performing the necessary normality and homogeneity tests. We intend to investigate this topic more deeply in future studies, including the author's thesis, where we plan to classify in detail the transformations used in specific subfields of agricultural sciences.

 A detailed classification of the reviewed studies, identifying the transformations used in specific subfields and their experimental situations, would enrich our article. The author continues this work as part of their thesis and plans to publish it in future articles. The researchers aimed to provide an overview of how the academic agricultural sciences community utilizes data transformation techniques. It has yielded valuable insights that we will further explore in subsequent studies.

 We appreciate the comments on the results presentation. We will correct the commas in the pie charts, replacing them with decimal points. Regarding the Sankey diagram, we chose to use it because it efficiently summarizes the trajectory of data transformations in the analyzed scientific research. The Sankey diagram allows us to present the information compactly and comprehensibly, avoiding the need for multiple bar charts, conditional probability charts, or flowcharts.

 A bar chart would be necessary for each variable presented, totaling eight bar charts to replace the two presented Sankey charts. If we presented the probability tree of the second Sankey chart, which has six variables with 2, 6, 6, 4, 4, and 4 possibilities, respectively, we would have a tree with 4068 branches (2*6*6*4*4*4=4608). We could also have made flowcharts with two variables at a time, totaling 12 flowcharts. Therefore, the Sankey chart summarizes a large amount of data without creating various tables, bar charts, or flowcharts. The reader can calculate any desired conditional probabilities since it presents the raw values.

 A more in-depth statistical analysis would be beneficial to identify patterns and underlying issues in the application of data transformations. However, this study aimed to provide an overview of data transformation practices in agricultural sciences. The author's thesis investigates the identification of detailed patterns and will address them in future studies.

 We value the feedback provided on the conclusion. We acknowledge that our current approach emphasizes presenting a method that facilitates systematic review research. However, our conclusion also addressed the results observed in our descriptive analysis, revealing the most used transformations in our sample. We emphasized the need for greater clarity and consistency in the documentation of the procedures used and the inconsistencies identified in applying the technique. For a more specific conclusion, we need to conduct more detailed studies of the subfields of agricultural sciences and the transformations used in each specific area. The author is investigating these issues in their doctoral thesis and plans to address them in future studies.

 The rationale and objectives of our study were to establish an overview of data transformation practices in agricultural sciences through a systematic review using an adapted screening method. This method facilitates research for researchers who wish to conduct systematic reviews, especially when the information of interest is present in the body of the article but not in the abstract and title. We will improve the last paragraph of the introduction to make this more precise and detailed.

 The statistical analysis conducted was adequate for this study's objectives, which were to provide an overview of data transformation practices. However, as mentioned earlier, we understand that additional statistical analyses could provide more insights, and this will be addressed in future studies.

 We based our study's conclusions on the presented results, which aimed to demonstrate an overview of data transformation practices in agricultural sciences. To achieve more detailed and assertive conclusions, we acknowledge the necessity of conducting a more in-depth study currently underway and scheduled for publication in future articles.

# Reviewer report: Reviewer response for version 1

**DOI:** 10.5256/f1000research.158652.r275872

**Published:** 2024-06-08

**Authors:** Pradip Kumar Sahu

**Affiliations:** 1 Department of Agricultural Statistics, Bidhan Chandra Krishi Viswa Vidyalaya, Nadia, West Bengal, India

This article based on 59 articles using transformation in ANOVA is very useful, it could have been still better if

 1) in addition to open access articles other articles are also considered

 2) in addition to percentage analysis use of other relevant statistical measures could have been more meaningful

 3) There is some confusion about sample of articles: written 70 in page 5 and 71 in page 8

 4) In page para5 of page 6 details of 88.56% articles have been provided what about rest 11.44% is not clear

 5) Information on how many articles have used the transformation properly is of great interest

 6) I would be interested to know about the results of test of agreement between the work of two reviewers

Are the rationale for, and objectives of, the Systematic Review clearly stated?

Yes

Is the statistical analysis and its interpretation appropriate?

Partly

If this is a Living Systematic Review, is the ‘living’ method appropriate and is the search schedule clearly defined and justified? (‘Living Systematic Review’ or a variation of this term should be included in the title.)

Not applicable

Are sufficient details of the methods and analysis provided to allow replication by others?

Yes

Are the conclusions drawn adequately supported by the results presented in the review?

Yes

Reviewer Expertise:

Basically I am an Agricultural Statistician having major areas of research as Regression analysis, Econometrics and Time series forecasting.

I confirm that I have read this submission and believe that I have an appropriate level of expertise to confirm that it is of an acceptable scientific standard, however I have significant reservations, as outlined above.

# Response

**Published:** 2024-07-10

**Authors:** Jhennifer dos Santos Nascimento

**Affiliations:** Statistics, Federal University of Vicosa, Viçosa, State of Minas Gerais, Brazil

Reviewer 1: Pradip Kumar Sahu, Department of Agricultural Statistics, Bidhan Chandra Krishi Viswa Vidyalaya, Nadia, West Bengal, India.

 Author responses to Reviewer comments are as follows:

**Reviewer Comment:**

 1) In addition to open access articles other articles are also considered

***Author Response: *** We appreciate the comment. We acknowledge that including closed-access articles would have enriched our study. However, limited financial resources restricted our access to paid articles, hampering our research. We used the articles available on the CAPES journal portal, which the Federal University of Viçosa pays for so that its students have access to various closed-access articles, and even so, there were articles that could not be downloaded. Specifically, we could not access 52 articles from our sample for financial reasons. Please refer to the link ( https://www-periodicos-capes-gov-br.ezl.periodicos.capes.gov.br/index.php/sobre/quem-somos.html) for more details about the CAPES journal portal.

**Reviewer Comment:**

 2) other relevant statistical measures could have been more significant besides the percentage analysis.

***Author Response: *** We agree that the inclusion of other statistical measures would enrich the analysis. We will add the raw values for each category presented in the images to Figure 1, complementing the percentage descriptive analysis. This way, the reader can obtain other statistics they deem relevant. If you have any specific statistical measures you would recommend including, we would be happy to consider them and incorporate them into our analysis.

**Reviewer Comment:**

 3) The sample of articles—70 on page 5 and 71 on page 8—needs to be clarified.

***Author Response: *** Thanks for pointing out this inconsistency. We corrected a typographical error on page 8. The correct number is 70, as presented on page 5.

**Reviewer Comment:**

 4) On page 6, details were provided for 88.56% of the articles, and the remaining 11.44% needs to be clarified.

***Author Response: *** The remaining 11.44% refers to articles that used power and box-cox transformations. We will clarify this in the text to avoid confusion.

**Reviewer Comment:**

 5) Information on how many articles correctly applied the transformation is fascinating.

 Information on how many articles correctly applied the transformation would indeed be fascinating.

***Author Response: *** Unfortunately, to do so, we would need the raw data from each article to replicate the analyses and determine whether the transformations were correctly applied. Since this data is unavailable, we cannot accurately assess the correctness of the transformations. So, our study focused on analyzing the application of transformations in the articles, not verifying their accuracy.

**Reviewer Comment:**

 6) I would like to know the results of the concordance test between the work of two reviewers.

***Author Response: *** Please provide more details about what you want to know, specifically about the concordance test between reviewers. This will allow us to address your question more precisely.
